# Estimating costs of periodontal treatment in public sector dental specialist clinics

**DOI:** 10.1186/1471-2458-14-S1-P11

**Published:** 2014-01-29

**Authors:** Tuti Mohd-Dom, Rasidah Ayob, Amrizal Muhammad-Nur, Noorlin Ishak, Mohd Rizal Abdul-Manaf, Syed Mohamed AlJunid, Ahmad Sharifuddin Mohd-Asari, Khairiyah Abdul-Muttalib

**Affiliations:** 1 United Nations University International Institute for Global Health (UNU-IIGH), 56000 Cheras, Kuala Lumpur, Malaysia; 2 Ministry of Health, Kuala Lumpur, Malaysia; 3 Jabatan Kesihatan Masyarakat, Pusat Perubatan Universiti Kebangsaan Malaysia, Jalan Yaacob Latif, Bandar Tun Razak, 56000 Cheras, Kuala Lumpur, Malaysia

## Background

Periodontitis is an established common chronic disease, yet its burden on health care costs remains largely neglected. The aim of this study was to estimate the cost of periodontal procedures in dental specialist clinics in the Malaysian public sector.

## Materials and methods

Five periodontal specialist clinics in the Ministry of Health were randomly selected. A list of periodontal procedures was identified by an expert group. Procedures were classified as diagnostics, non-surgical periodontics and periodontal surgeries. Costing for each procedure was primarily activity-based to measure cost of dental equipment, consumables and labour cost (average treatment time). Expenditures for administration, utilities and maintenance at clinics used unit cost calculations employed from step-down approach. A total of 165 patients newly diagnosed with periodontitis were followed up for one year to determine the cost of managing these patients during the said period.

## Results

A total examination package costed USD38, but separate diagnostics procedures such as full-mouth periodontal assessment, electric pulp test, intraoral and panoramic radiographs costed an average of USD23. For nonsurgical procedures, full-mouth supragingival scaling costed USD68 and full-mouth subgingival debridement costed USD135, higher if with systemic/ local delivery antibiotics. Costs for desensitisation, occlusal adjustments (with selective grinding) and splinting were USD41, USD42 and USD50, respectively. Surgical procedures cost an average of USD294 for resective surgeries and USD613 for regenerative surgeries. The total provider costs for managing periodontitis patients for one year was estimated to be USD805. The costs borne for patients requiring surgeries (USD1,512) was about three-fold (t-test, P<0.001) compared to those who required only nonsurgical periodontal treatment (USD540).

## Conclusions

Specialist dental treatment for periodontitis patients in the public sector is costly. Cost estimates obtained from this study are useful for estimating economic burden of specialist periodontitis management at the national level, as well as providing the basis for economic evaluation of the specialist periodontal care programme.

